# Supplementary material for: MetaRibo-Seq measures translation in microbiomes
Source: Nat Commun. 2020 Jun 29;11:3268. doi: 10.1038/s41467-020-17081-z (PMC7324362; doi:10.1038/s41467-020-17081-z)
Supplement: Supplementary file 10 — Supplementary Data 7 [file 41467_2020_17081_MOESM10_ESM.zip › File2/Confidence_High_Taxonomy/57028_out.krona.html]

Javascript must be enabled to view this page.

members
magnitude
magnitudeUnassigned
count
unassigned
taxon
rank

57028\_out

77

2
77
superkingdom

77
1239
phylum

77
186801
class

order
77
186802

family
76
186803

genus
75
841


SRS011084\_contig\_number\_13388SRS013158\_contig\_number\_contig-100\_90.40107SRS013940\_contig\_number\_13339SRS013951\_contig\_number\_14500SRS014287\_contig\_number\_5146SRS014412\_contig\_number\_contig-100\_1214.119906SRS014613\_contig\_number\_19988SRS014855\_contig\_number\_12097SRS014923\_contig\_number\_12603SRS015065\_contig\_number\_26419SRS015190\_contig\_number\_14186SRS015264\_contig\_number\_17213SRS015431\_contig\_number\_40948SRS015578\_contig\_number\_15092SRS015579\_contig\_number\_23769SRS015890\_contig\_number\_13913SRS015960\_contig\_number\_21687SRS016267\_contig\_number\_8725SRS016335\_contig\_number\_contig-100\_912.254423SRS017821\_contig\_number\_11500SRS018623\_contig\_number\_12271SRS018984\_contig\_number\_29348SRS019397\_contig\_number\_11616SRS019787\_contig\_number\_8500SRS021484\_contig\_number\_30860SRS022137\_contig\_number\_12764SRS024388\_contig\_number\_10114SRS024549\_contig\_number\_4552SRS043001\_contig\_number\_contig-100\_310.68987SRS043411\_contig\_number\_3179SRS043701\_contig\_number\_17151SRS045739\_contig\_number\_9690SRS049900\_contig\_number\_20453SRS050422\_contig\_number\_11603SRS050925\_contig\_number\_27862SRS050941\_contig\_number\_20359SRS052027\_contig\_number\_15684SRS053335\_contig\_number\_14892SRS054956\_contig\_number\_2731SRS055017\_contig\_number\_36431SRS056273\_contig\_number\_21701SRS058070\_contig\_number\_13127SRS063040\_contig\_number\_5972SRS065504\_contig\_number\_37142SRS075341\_contig\_number\_22780SRS075716\_contig\_number\_4680SRS076756\_contig\_number\_contig-100\_404.122911SRS077454\_contig\_number\_10968SRS077641\_contig\_number\_23095SRS098644\_contig\_number\_32776SRS098827\_contig\_number\_contig-100\_119.88290SRS098881\_contig\_number\_23350SRS1041130\_contig\_number\_5282SRS104485\_contig\_number\_2599SRS104975\_contig\_number\_15001SRS105153\_contig\_number\_16079SRS1055022\_contig\_number\_19181SRS1055034\_contig\_number\_10057SRS142503\_contig\_number\_16618SRS142781\_contig\_number\_11284SRS143070\_contig\_number\_contig-100\_51.109872SRS143417\_contig\_number\_24589SRS143722\_contig\_number\_14651SRS143780\_contig\_number\_13097SRS144362\_contig\_number\_41151SRS144537\_contig\_number\_5713SRS147919\_contig\_number\_4675SRS148091\_contig\_number\_11825SRS148159\_contig\_number\_41851SRS148424\_contig\_number\_30625SRS148784\_contig\_number\_23214SRS149181\_contig\_number\_21184SRS149879\_contig\_number\_17913SRS893341\_contig\_number\_755SRS893378\_contig\_number\_11948
species
75
166486

189330
1
genus


SRS019068\_contig\_number\_4063
species
1
39486

186806
1
family

1730
1
genus

1
1262883
species

SRS056519\_contig\_number\_10646
